# Dietary patterns, nutrition, and risk of breast cancer: a case-control study in the west of Iran

**DOI:** 10.4178/epih.e2019003

**Published:** 2019-01-24

**Authors:** Behjat Marzbani, Javad Nazari, Farid Najafi, Behnaz Marzbani, Sara Shahabadi, Mahin Amini, Mehdi Moradinazar, Yahya Pasdar, Ebrahim Shakiba, Saeed Amini

**Affiliations:** 1 Health Education and Promotion Group, Vice Chancellor for Health Affairs, Kermanshah University of Medical Sciences, Kermanshah, Iran; 2 Department of Pediatric, School of Medicine, Arak University of Medical Sciences, Arak, Iran; 3 Research Center for Environmental Determinants of Health, School of Public Health, Kermanshah University of Medical Sciences, Kermanshah, Iran; 4 Family and School Health Group, Health Network of Kermanshah, Vice Chancellor for Health Affairs, Kermanshah University of Medical Sciences, Kermanshah, Iran; 5 Nutritional Sciences Department, School of Public Health, Kermanshah University of Medical Sciences, Kermanshah, Iran; 6 Department of Clinical Biochemistry, Medical School, Kermanshah University of Medical Sciences, Kermanshah, Iran; 7 Department of Health Services Management, Arak University of Medical Sciences, Arak, Iran

**Keywords:** Diet, Breast cancer, Case-control studies, Iran

## Abstract

**OBJECTIVES:**

Unhealthy dietary patterns are the most important changeable risk factors for breast cancer. The aim of this study was to assess the relationship between dietary patterns and the risk of breast cancer among under-50 year women in the west of Iran.

**METHODS:**

All women under 50 years old with pathologically confirmed breast cancer between 2013 and 2015 who were referred to oncology clinics in the west of Iran, and 408 under-50 women referred to other outpatient clinics who were without breast or other cancers at the time of the study and 2 years later were selected as the control group. The data were collected using the middle-aged periodical care form of the Iranian Ministry of Health and analyzed using univariate and multivariate logistic regression in Stata.

**RESULTS:**

The most powerful risk factor for breast cancer was fried foods; the odds ratio of consuming fried foods more than once a month for breast cancer was 4.5 (95% confidence interval, 2.1 to 9.4). A dose-response model indicated that increasing vegetable and fruit consumption up to 90 servings per month decreased the odds of breast cancer, but consuming more than 90 servings per month increased the risk.

**CONCLUSIONS:**

Inadequate consumption of vegetables and consumption of soft drinks, industrially produced juices, fried foods, and sweets were identified as risk factors for breast cancer. In response to these findings, it is necessary to raise awareness and to provide education about healthy diets and the need to change unhealthy dietary patterns.

## INTRODUCTION

Cancer is one of the most important causes of death throughout the world [1]. The World Health Organization (WHO) has predicted that cancer-related deaths will affect 24 million people by 2035. Breast cancer is one of the most common types of cancer, and it is the leading cause of cancer deaths among women in both developing and developed countries [1-3]. The WHO has reported that the incidence of breast cancer is increasing by between 1.8% and 2.0% annually around the world [4]. During 2005 to 2020 nearly 26% increase in breast cancer cases occurs and the increase is expected to be higher in developing countries [1,2].

The epidemiological model of breast cancer in Iran is similar to that of other east Mediterranean and developing countries [2]. On the basis of the cancer registration report of the Iranian Ministry of Health, breast cancer, with an age-standardized incidence rate of 28.2%, is the most prevalent cancer among Iranian women. The mean age of breast cancer patients is 10 to 15 years lower in Iran than in developed countries and the global mean age; previous studies have indicated that the mean age of breast cancer patients in Iran is between 40 years old to 50 years old [5,6]. Mousavi et al. [7] reported that 23% of cases of breast cancer in Iranians were diagnosed in patients younger than 40 years old, 70% of whom were diagnosed in the progressive stage of the disease. However, early diagnosis of the disease, especially in the first stages, is very important for reducing mortality from breast cancer [6].

The American Cancer Society has identified several risk factors for breast cancer, including age, gender, family history, early menstruation, hysterectomy, fibrocystic history, family history of uterine and ovarian cancers, and history of radiation into the chest; the majority of these risk factors are unchangeable [8]. However, other studies have identified changeable risk factors for breast cancer, including dietary patterns. A significant relationship exists between different dietary patterns and the risk of breast cancer [9,10], and correcting dietary patterns can prevent one-third of the morbidity and mortality of breast cancer [11].

Few studies have assessed the changeable risk factors of breast cancer in Iran, especially the role of dietary patterns [12,13]. Regional variation in the incidence of breast cancer and the numerous risk factors of breast cancer have necessitated studies on dietary patterns as an effective changeable factor for preventing breast cancer. This study assessed the relationship between dietary patterns and the risk of breast cancer.

## MATERIALS AND METHODS

### Study population

**Figure 1.**
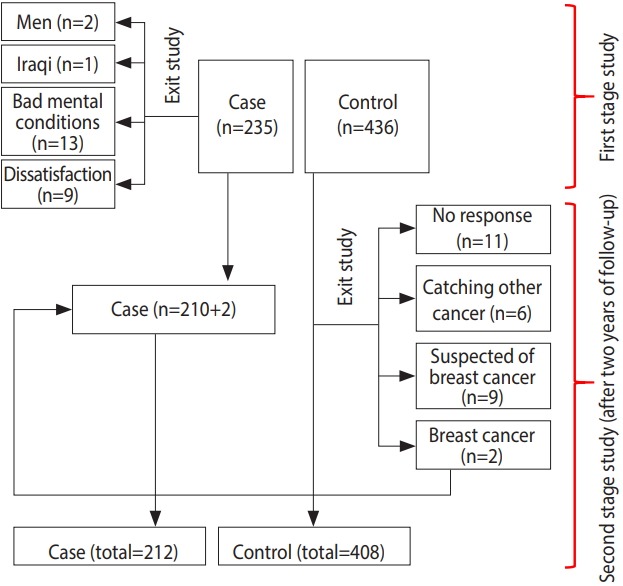
Flowchart of the research process.

This case-control study was performed among 620 women (212 cases and 408 controls) in Kermanshah Province, in the west of Iran. In the first phase, all women under 50 years old with pathologically confirmed breast cancer (235 cases) between 2013 and 2015 who were referred to the radiation therapy, oncology, and chemotherapy clinics of the cancer diagnosis referral center in the west of Iran (Imam Reza Hospital) were selected as the cases. As Figure 1 indicates, 25 patients were excluded, including 1 (4.0%) Iraqi because of language difficulties, 2 (8.0%) men, 13 (52.0%) persons who did not feel that they were in a suitable spiritual situation to participate in the study because of the recent diagnosis of their disease, and 9 (36%) persons who declined to participate in the study. The control group was then selected randomly among patients referred to other wards of the Imam Reza Hospital. For each case, 2 controls were enrolled, with the criteria of not currently having breast cancer and lacking a previous history of any cancer.

In the second phase, the case and control patients were matched by age, with a 4-year range. Due to the possibility of breast cancer incidence in the control group, all persons in the control group were followed for 2 years. During this time period, 28 persons were excluded from the control group: 6 (21.4%) because of having other cancers, 9 (32.2%) persons because of suspicion of breast cancer based on symptoms, 11 (39.2%) persons due to lack of a response, and 2 (7.1%) persons because of pathologically confirmed breast cancer. These 2 persons were added to the case group (Figure 1).

### Data collection

Data were collected through verbal interviews, a review of medical records, and telephone follow-ups. To collect data on additional demographic and anthropometric variables, the middle-aged integrated health care form of the Iranian Ministry of Health was used to assess dietary patterns [14]. This questionnaire is used routinely by Iranian Ministry of Health to assess individuals’ diet. The validity and reliability of this questionnaire has been confirmed by previous studies [15]. It includes 11 questions regarding nutritional status and dietary habits (consuming dairy products, vegetables, fruits, fast foods, soft drinks, industrially produced juices, fats and solid oils, salt, fried foods, sweets, and low-nutritional-value snacks). The amount of standardized servings usually consumed in a day was used to measure the consumption of dairy products, vegetables, and fruits.

As indicated in Table 1, a favorable amount of daily consumption of dairy products is 2-3 servings per day, and the corresponding values for fruits and vegetables are 2-4 servings and 3-5 servings, respectively. The survey inquired whether the participants had consumed fast foods, soft drinks, industrially produced juices, salt, fried and high-fat foods, sweets, and low-nutritional-value snacks. For these products, responses of never or once a month were considered favorable, while responses of 2 times or more per month were considered unfavorable. Consumption of vegetable-based oils was considered favorable, while consumption of solid, semi-solid, animal, or a mixture of liquid and solid fats was considered unfavorable (Table 1).

### Ethical statement

Before collecting the data, informed consent was obtained from the participants. All ethical considerations, including obtaining verbal consent, were approved by the Ethical Committee of Kermanshah University of Medical Sciences.

### Data analysis

The relationships between the variables were assessed using univariate and multivariate logistic regression. Variables with a p-value <0.3 in the univariate analysis were entered into the multivariate model. Then, using forward or backward methods, variables with a p-value more than 0.05 were removed from the model.

Fractional polynomials were used to quantify the effects of patterns of food consumption on the odds of breast cancer. Fractional polynomials are an alternative to regular polynomials that provide flexible parameterization for continuous variables. In this method, the effects of demographic variables and body mass index (BMI) on breast cancer risk were first controlled. Then, the impact of food consumption models was assessed. Fewer than 1% of the data points were missing, and missing data points were deleted from the study. All analyses were performed using Stata version 14.1 (StataCorp., College Station, TX, USA) with 95% confidence intervals (CIs).

## RESULTS

In total, 620 women under 50 years old (212 cases and 408 controls) were studied. Their mean age was 41.5±6.2 years and 39.5± 7.1 years for the case and control groups, respectively. No statistically significant relationship was found between breast cancer and place of residence, marital status, employment status, insurance coverage, or BMI in the univariate and multivariate analyses. The odds ratio (OR) of breast cancer increased with higher levels of education, so that the OR of breast cancer in women with primary and secondary schooling was 2.2 in comparison to illiterate women (OR, 2.2; 95% CI, 1.2 to 4.0) and the OR for women with university education was 2.8 in comparison to illiterate women (OR, 2.8; 95% CI, 1.3 to 5.9).

In the univariate analysis, breast cancer risk decreased with increasing fruit consumption, and in the univariate and multivariate analyses, breast cancer risk decreased with increasing vegetable consumption. Therefore, the OR of breast cancer associated with consumption of 2-3 servings of vegetables per month was 2.8 in comparison to daily consumption (OR, 2.8; 95% CI, 1.7 to 4.5).

The OR of breast cancer in women who consumed soft drinks (OR, 2.8; 95% CI, 1.9 to 4.3), industrially produced juices (OR, 2.7; 95% CI, 1.1 to 6.5) and solid oils (OR, 1.9; 95% CI, 1.3 to 3.0) more than once a month was significantly elevated relative to their counterparts who did not consume those substances or did so no more than once a month.

The most powerful risk factor for breast cancer in the univariate and multivariate analyses was fried foods, even though the magnitude of this relationship decreased after controlling for other variables. The OR of breast cancer in women consuming fried foods more than once a month was 4.5 in comparison to women who consumed fried foods never or once a month (95% CI, 2.1 to 9.4).

A significant relationship was found between sweets consumption and the odds of breast cancer. The OR of breast cancer in women who consumed sweets more than a month was 2.6 in comparison with women whose sweets consumption was in the favorable range (95% CI, 1.7 to 3.9) (Table 2).

**Figure 2.**
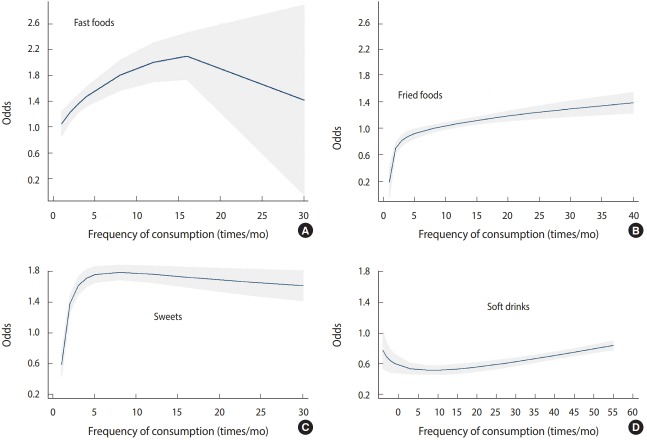
Dose-response relationships of (A) fast food, (B) fried foods, (C) sweets, and (D) soft drinks in terms of the frequency of consumption per month with the odds of breast cancer based on fractional polynomials adjusted for demographic variables including age, gender, education level, and body mass index.

After controlling for demographic variables, including age, education level, and BMI, a non-linear relationship was found between the odds of breast cancer and consumption of food materials. In particular, the consumption of fast foods and soft drinks increased the odds of breast cancer (Figure 2).

**Figure 3.**
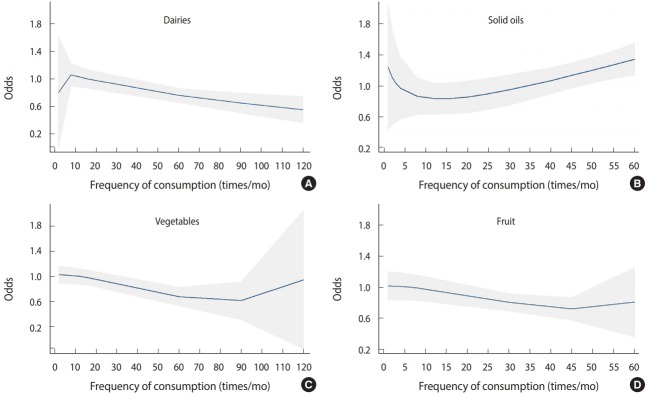
The odds of breast cancer associated with the consumption of (A) dairy products, (B) fats and oils, (C) vegetables, and (D) fruits in terms of the frequency of consumption per month.

The relationship of fruit and vegetable consumption with breast cancer was analyzed using the same model. The odds of breast cancer decreased as fruit and vegetable consumption increased to 90 units per month, but increased at higher levels of consumption (Figure 3).

## DISCUSSION

The risk of breast cancer was found to be higher in women who consumed soft drinks more than once a month than in those who did not consume soft drinks or did so once a month. It has been previously reported that the consumption of soft drinks significantly increases the odds of breast cancer. Soft drinks, since they are calorically dense, lead to increases in BMI, obesity, and insulin resistance, which are mediators of cancer risk [11,15]. The dose-response pattern indicated that consuming soft drinks more than 12 times a month increased the odds of breast cancer. However, the odds of having breast cancer in women who consumed soft drinks 10-12 times per month was even lower than in those who never consumed soft drinks. This may have been because of other unassessed chronic diseases or because of a possible preventive role of consuming small amounts of soft drinks, similar to the effect that has been observed for alcohol. Further studies are needed to clarify the underlying causality.

In a meta-analysis of 55 articles, Boyle et al. [16] assessed the relationship between soft drink consumption and various cancers, including breast cancer, and concluded that consumption of various levels of soft drinks did not increase the risk of the studied cancers. A reason for this may have been the concomitant consumption of high-fat foods along with soft drinks.

Dong et al. [17], in a meta-analysis of prospective cohort studies, assessed the relationship between dairy consumption and risk of breast cancer. They reported that the odds of breast cancer decreased with increasing dairy consumption. The current study found that after controlling for confounding variables, there was no significant relationship between dairy consumption and the odds of breast cancer. However, according to the dose-response pattern, the odds of breast cancer decreased with increasing dairy consumption. Differences in the fat composition of dairy products in different studies may account for discrepancies in the results across studies, as it has been reported that the consumption of low-fat dairy products decreases the odds of breast cancer and vice versa [12]. Farvid et al. [18] indicated that high-fat dairy products increased the risk of estrogen and progesterone receptor negative tumors.

On the basis of the dose-response graph, although the odds of breast cancer increased with the frequency of fast food consumption, the relationship was not significant. The odds of breast cancer associated with consuming fast foods more than 15 times a month showed high variation and a wide confidence interval, which may have been because of the low sample size. Therefore, it did not show statistical significance. Michels et al. [19], in a study of 89,887 women, concluded that this relationship was significant. Regarding the cause, they have reported that sodium nitrate in processed meats, such as sausage, burgers, and pizza, is converted to nitrosamine, which is a known carcinogen. Researchers from Poland have reported that the odds of breast cancer increased 3 times with daily consumption of fast foods [20].

The most important result of the current study is the highly significant relationship found between consumption of fried foods and the odds of breast cancer, as the odds of breast cancer in women who frequently consumed fried foods were 4.5 times higher than among their counterparts. A study by Agurs-Collins et al. [21] regarding dietary patterns and breast cancer risk in women reported similar findings. In this regard, it is necessary to perform studies on the methods of frying, the types of fried foods, the temperature used in frying, and the duration of frying. Because more than half of the participants in this study consumed solid and semi-solid fats, the effect of fried food on breast cancer in this study was higher than in another similar study [22].

The results indicated that the odds of breast cancer in women who consumed unfavorable fats more than once a month (solid, semi-solid, and animal fats, or a mixture of liquid and solid fats) was higher than women who consumed such fats never or once a month. Holmes & Willett [23] reported that animal fat consumption was linked to the risk of breast tumors. However, Sieri et al. [24] did not report a significant relationship between fat consumption and the risk of breast cancer. It can be concluded that the consumption of different types of fats and oils have different effects on the odds of breast cancer [25].

The dose-response pattern indicated that more frequent sweets consumption increased the odds of breast cancer, with a steep slope. Of note, in a study conducted in Australia, Harray et al. [26] found that people who paid more attention to their health situation consumed smaller amounts of junk foods.

A significant direct relationship was found between the odds of breast cancer and education level. Other studies confirm this result [27,28]. However, other studies have reported an opposite pattern. For example, education level was proposed as a protective factor in an Indian study. The authors of that study stated that the effects of confounding factors such as socioeconomic status explained the protective effect of education, as higher levels of education indirectly affect dietary patterns [29,30].

The consumption of vegetables is recommend because of their antioxidant properties. An inverse relationship was found between vegetable consumption and the odds of breast cancer. In other words, the odds of breast cancer increased as vegetable consumption decreased. For example, the odds of breast cancer increased when vegetable consumption decreased from daily consumption to weekly or monthly consumption. Boffetta et al. [31] obtained similar results in a study that aimed to compare fruit and vegetable intake and the risk of cancer among the European Union countries. In the present study, the analysis of the dose-response pattern indicated that the odds of breast cancer decreased as fruit and vegetable consumption increased to 90 servings per month, but then increased with higher levels of consumption. This may have been caused by other factors, such as an increased caloric intake, exceeding the body’s requirements.

No significant relationship was found between the odds of breast cancer and place of residence. In this regard, previous studies have indicated that differences in the risk of breast cancer according to place of residence are more likely to stem from differences in diet patterns and personal habits than from environmental factors. Additionally, as the lifestyle of people living in rural and urban regions has become more similar, it is expected that differences in the rate of breast cancer between rural and urban regions will decrease in the future [32].

No significant relationship was found between the odds of breast cancer and marital status. However, international studies have indicated that single women have a higher risk of breast cancer than married women. Overall, on one hand, it might seem that married, divorced, and widowed women have no inherent differences in their risk of breast cancer compared with single women, and that the apparent protective effect of marriage may be because of the age of their first pregnancy and childbirth. Additionally, older age at marriage leads to delays in the first pregnancy and childbirth, which are a risk factor for breast cancer. On the other hand, single women less frequently visit doctors, which may lead to late diagnosis and treatment [6,33].

BMI is known to affect the risk of breast cancer, but no significant relationship was found in the current study. This is in accordance with the study of Alim & Kiziltan [10], but Galukande et al. [32] reported an inverse relationship between BMI and the probability of breast cancer; however, this may have been because of weight loss in the case group in the time before the disease being diagnosed. Many studies have reported a positive and significant relationship between obesity and high BMI with breast cancer risk. Estrogen release from fat cells has been proposed as the cause for this relationship [34,35]. After the teenage years, the risk of breast cancer increases with BMI; therefore, maintaining one’s weight in the normal range is necessary to prevent breast cancer [36].

Age is known to be the most important and challenging risk factor for breast cancer, as the risk of breast cancer dramatically increases with age [37]. The current study likewise indicated that there was a significant and direct relationship between increasing age and the odds of breast cancer. However, based on the conflicting results of studies of the prognosis of breast cancer in young women [38].

Although this study has some limitations, such as not assessing other risk factors such as hormonal medications, the sensitivity and specificity of the logistic model are indicative of the adequacy of the model and the studied variables. Another limitation is the method of measuring nutrition status; although the validity and reliability of the questionnaire have been confirmed, it is known that responses to nutrition questions include both over-reporting and under-reporting. Trained interviewers who were thoroughly familiar with the nutrition questionnaire were used to decrease this type of error. We sought to minimize the inherent risk of bias associated with case-control studies by assessing the treatment history and 2-year follow-up of the control group.

In summary, increasing age, higher education levels, inadequate consumption of vegetables, and consumption of soft drinks, industrially produced juices, fried foods, and sweets were identified as risk factors for breast cancer among women under 50. Therefore, it is essential to raise awareness and to disseminate education about a healthy diet and how to change unhealthy dietary patterns. Certain risk factors, such as BMI, place of residence, lack of daily consumption of dairy products and fruits, and consumption of salt and fast foods were not found to be related with the risk of breast cancer in this study, but have been in other studies, suggesting that it is necessary to assess these factors in greater depth.

The main measures to strengthen cultural support for consuming a healthy diet that would reduce the risk of breast cancer include acceptance of responsibility by political leaders from the capital to the most remote villages and funding initiatives that promote healthy dietary patterns. More local measures include carrying out national and subnational campaigns such as public walking initiatives; educational sessions; designing brochures, posters and banners and distributing them in cities and villages; and showing appreciation for the healthcare workers who are active in this regard.

## Figures and Tables

**Table 1.** Measurements of servings and definitions of favorable amounts of consumption of food materials

Food material	Amount of each serving	Favorable amount (recom- mended consumption)
Dairy	A cup of milk or yogurt = 45 to 60 g of typical cheese (one ounce) = one-fourth of a cup of whey = 2 cups of dough (Iranian drink)= one and a half cups of vanilla ice cream	2-3 serving/d
Vegetables	A cup of raw leafy vegetables = half a cup of cooked or raw chopped vegetables = half a cup of starchy vegetables (green peas, beans, corn, green beans and carrots) = a medium- sized tomato; carrots; cucumbers; raw onions	3-5 serving/d
Fruits	(A medium-sized) apple, banana, orange, pear, peach, kiwi, nectarine = half a cup of small fruits such as berries, grapes and pomegranates = 2 tangerines = half of a grapefruit = 12 cherries = 2 plums or dates or fresh figs = 4 medium-sized apricots = 300 grams of differ- ent melons = half a cup of cooked fruit or a compote = one-fourth of a cup of dried fruit or nuts = three-quarters of a cup of fresh and natural juice	2-4 serving/d
Fast food	Sausage; pizza; fast food; Mexican corn; fried potatoes; salty, smoked and canned meat	Never or once a month
Soft drinks	Carbonated drinks (cola, various drinks, non-alcoholic beer)	Never or once a month
Industrially produced juices	Various industrially produced juices	Never or once a month
Fats and oils	Solid, semi-solid, or animal fat or a mixture of liquid or solid fat	Never or once a month
Salt	Use of table salt from a salt shaker	Never or once a month
Fried foods	Fried with high fat	Never or once a month
Sweets	Various sweets, chocolate, and sugar	Never or once a month
Low-nutritional-value snacks	Chips, puffs	Never or once a month

**Table 2.** Univariate and multivariate analysis of dietary patterns and risk of breast cancer

Variable		Case/control	Crude OR (95% CI)	Adjusted OR (95% CI)
Age (yr)	≤30	14/69	1.0 (reference)	1.0 (reference)
	31-40	70/146	2.4 (1.2, 4.5)	-
	41-50	128/193	3.3 (1.7, 6.0)	-
Education	Illiterate	28/78	1.0 (reference)	1.0 (reference)
	Less than a high school diploma	107/167	1.7 (1.0, 2.9)	2.2 (1.2, 4.0)
	High school diploma	41/79	1.4 (0.8, 2.5)	2.2 (1.1, 4.5)
	College/university	36/84	1.9 (0.6, 2.1)	2.8 (1.3, 5.9)
Location	Urban	170/349	1.0 (reference)	-
	Rural	42/59	1.4 (0.9, 2.2)	-
Marital status	Married	175/332	1.0 (reference)	-
	Single	27/56	0.9 (0.5, 1.4)	-
	Separated/divorced/widowed	10/20	0.9 (0.4, 2.0)	-
Job	Housekeeper	184/355	1.0 (reference)	-
	Government employee	16/33	0.9 (0.5, 1.7)	-
	Private-sector employee	12/20	1.1 (0.5, 2.4)	-
Insurance coverage	Yes	207/400	1.0 (reference)	-
	No	5/8	1.2 (0.3, 3.7)	-
BMI (kg/m^2^)	<24.9	63/140	1.0 (reference)	-
	24.9-29.9	99/166	1.3 (0.8, 1.9)	-
	29.9-34.9	35/84	0.9 (0.5, 1.5)	-
	>34.9	15/18	1.8 (0.8, 3.9)	-
Dairy consumption	Daily	114/275	1.0 (reference)	-
	2-3 serving/wk	43/50	2.0 (1.3, 3.2)	-
	2-3 serving/mo	55/83	1.5 (1.0, 2.3)	-
Consumption of vegetables	Daily	52/191	1.0 (reference)	1.0 (reference)
	2-3 serving/wk	58//85	2.5 (1.5, 3.9)	1.7 (1.0, 2.9)
	2-3 serving/mo	102/132	2.8 (1.9, 4.2)	2.8 (1.7, 4.5)
Consumption of fruits	Daily	133/317	1.0 (reference)	-
	2-3 serving/wk	56/57	2.3 (1.5, 3.5)	-
	2-3 serving/mo	23/34	1.6 (0.9, 2.8)	-
Fast food	Favorable	43/38	1.0 (reference)	-
	Unfavorable	169/370	2.4 (1.5, 3.9)	-
Soft drinks	Favorable	127/113	1.0 (reference)	1.0 (reference)
	Unfavorable	85/295	3.9 (2.7, 5.5)	2.8 (1.9, 4.3)
Industrially produced juices	Favorable	19/12	1.0 (reference)	1.0 (reference)
	Unfavorable	193/396	3.2 (1.5, 6.8)	2.7 (1.1, 6.5)
Fats and oils	Favorable	151/220	1.0 (reference)	1.0 (reference)
	Unfavorable	61/188	2.1 (1.4, 3.0)	1.9 (1.3, 3.0)
Salt	Favorable	130/227	1.0 (reference)	-
	Unfavorable	82/181	1.2 (0.9, 1.7)	-
Fried foods	Favorable	202/288	1.0 (reference)	1.0 (reference)
	Unfavorable	10/120	8.4 (4.3, 16.4)	4.5 (2.1, 9.4)
Sweets	Favorable	117/101	1.0 (reference)	-
	Unfavorable	94/307	3.7 (2.6, 5.3)	2.6 (1.7, 3.9)
Low-nutritional-value snacks	Favorable	36/27	1.0 (reference)	-
	Unfavorable	176/381	2.8 (1.6, 4.9)	-
Goodness of fit model				
Sensitivity	55.9			
Specificity	86.8			
Accuracy	76.3			

OR, odds ratio; CI, confidence interval.

## References

[1] Torre LA, Bray F, Siegel RL, Ferlay J, Lortet-Tieulent J, Jemal A (2015). Global cancer statistics, 2012. CA Cancer J Clin.

[2] Roohparvarzade N (2014). Prevalence of risk factors for breast cancer in women (20 to 69 years old) in Isfahan 2012-2013. Iran J Breast Dis.

[3] DeSantis C, Siegel R, Bandi P, Jemal A (2011). Breast cancer statistics, 2011. CA Cancer J Clin.

[4] Walker RA, Lees E, Webb MB, Dearing SJ (1996). Breast carcinomas occurring in young women (<35 years) are different. Br J Cancer.

[5] Motie MR, Besharat S, Torkjazi R, Shojaa M, Besharat M, Keshtkar A (2011). Modifiable risk of breast cancer in northeast iran: hope for the future. a case-control study. Breast Care (Basel).

[6] Tehranian N, Shobeiri F, Pour FH, Hagizadeh E (2010). Risk factors for breast cancer in Iranian women aged less than 40 years. Asian Pac J Cancer Prev.

[7] Mousavi SM, Montazeri A, Mohagheghi MA, Jarrahi AM, Harirchi I, Najafi M (2007). Breast cancer in Iran: an epidemiological review. Breast J.

[8] Kelsey JL, Berkowitz GS (1988). Breast cancer epidemiology. Cancer Res.

[9] Kotepui M (2016). Diet and risk of breast cancer. Contemp Oncol (Pozn).

[10] Alim NE, Kiziltan G (2016). Assessment of risk factors of obesity and diet on breast cancer in Ankara, Turkey. Pak J Med Sci.

[11] Brennan SF, Cantwell MM, Cardwell CR, Velentzis LS, Woodside JV (2010). Dietary patterns and breast cancer risk: a systematic review and meta-analysis. Am J Clin Nutr.

[12] Kojima R, Okada E, Ukawa S, Mori M, Wakai K, Date C (2017). Dietary patterns and breast cancer risk in a prospective Japanese study. Breast Cancer.

[13] Butler LM, Wu AH, Wang R, Koh WP, Yuan JM, Yu MC (2010). A vegetable-fruit-soy dietary pattern protects against breast cancer among postmenopausal Singapore Chinese women. Am J Clin Nutr.

[14] Allameh M, Farahani A, Tabatabaee M (2016). Integrated care guidelines for middle aged health in Iran. http://www.behdasht.gov.ir/.

[15] Lim S, Zoellner JM, Lee JM, Burt BA, Sandretto AM, Sohn W (2009). Obesity and sugar-sweetened beverages in African-American preschool children: a longitudinal study. Obesity (Silver Spring).

[16] Boyle P, Koechlin A, Autier P (2014). Sweetened carbonated beverage consumption and cancer risk: meta-analysis and review. Eur J Cancer Prev.

[17] Dong JY, Zhang L, He K, Qin LQ (2011). Dairy consumption and risk of breast cancer: a meta-analysis of prospective cohort studies. Breast Cancer Res Treat.

[18] Farvid MS, Eliassen AH, Cho E, Chen WY, Willett WC (2018). Dairy consumption in adolescence and early adulthood and risk of breast cancer. Cancer Epidemiol Biomarkers Prev.

[19] Michels KB, Willett WC, Rosner BA, Manson JE, Hunter DJ, Colditz GA (1996). Prospective assessment of breastfeeding and breast cancer incidence among 89,887 women. Lancet.

[20] Plagens-Rotman K, Piskorz-Szymendera M, Chmaj-Wierzychowska K, Pieta B (2017). Breast cancer - analysis of the selected risk factors. Eur J Gynaecol Oncol.

[21] Agurs-Collins T, Rosenberg L, Makambi K, Palmer JR, Adams-Campbell L (2009). Dietary patterns and breast cancer risk in women participating in the Black Women’s Health Study. Am J Clin Nutr.

[22] Bahrami G, Bahrami S, Bahrami MT, Pasdar Y, Rezaei M, Darbandi M (2015). The trans fatty acid content of snacks offered in Kermanshah. Int J Health Life Sci.

[23] Holmes MD, Willett WC (2004). Does diet affect breast cancer risk?. Breast Cancer Res.

[24] Sieri S, Krogh V, Ferrari P, Berrino F, Pala V, Thiébaut AC (2008). Dietary fat and breast cancer risk in the European Prospective Investigation into Cancer and Nutrition. Am J Clin Nutr.

[25] Thiébaut AC, Chajès V, Gerber M, Boutron-Ruault MC, Joulin V, Lenoir G (2009). Dietary intakes of omega-6 and omega-3 polyunsaturated fatty acids and the risk of breast cancer. Int J Cancer.

[26] Harray AJ, Boushey CJ, Pollard CM, Panizza CE, Delp EJ, Dhaliwal SS (2017). Perception v. actual intakes of junk food and sugar-sweetened beverages in Australian young adults: assessed using the mobile food record. Public Health Nutr.

[27] Kamath R, Mahajan KS, Ashok L, Sanal TS (2013). A study on risk factors of breast cancer among patients attending the tertiary care hospital, in udupi district. Indian J Community Med.

[28] Naieni KH, Ardalan A, Mahmoodi M, Motevalian A, Yahyapoor Y, Yazdizadeh B (2007). Risk factors of breast cancer in north of Iran: a case-control in Mazandaran Province. Asian Pac J Cancer Prev.

[29] Besharat S, Motie MR, Besharat M, Roshandel G (2011). Breast cancer risk factors in women of Golestan Province in Iran: a case-control study. Iran J Obstet Gynecol Infertil.

[30] Das S, Sen S, Mukherjee A, Chakraborty D, Mondal PK (2012). Risk factors of breast cancer among women in eastern India: a tertiary hospital based case control study. Asian Pac J Cancer Prev.

[31] Boffetta P, Couto E, Wichmann J, Ferrari P, Trichopoulos D, Bueno-de-Mesquita HB (2010). Fruit and vegetable intake and overall cancer risk in the European Prospective Investigation into Cancer and Nutrition (EPIC). J Natl Cancer Inst.

[32] Galukande M, Wabinga H, Mirembe F, Karamagi C, Asea A (2016). Breast cancer risk factors among Ugandan women at a tertiary hospital: a case-control study. Oncology.

[33] Ebrahimi M, Vahdaninia M, Montazeri A (2002). Risk factors for breast cancer in Iran: a case-control study. Breast Cancer Res.

[34] Protani M, Coory M, Martin JH (2010). Effect of obesity on survival of women with breast cancer: systematic review and meta-analysis. Breast Cancer Res Treat.

[35] Goodwin PJ, Stambolic V (2015). Impact of the obesity epidemic on cancer. Annu Rev Med.

[36] Hsieh CC, Trichopoulos D, Katsouyanni K, Yuasa S (1990). Age at menarche, age at menopause, height and obesity as risk factors for breast cancer: associations and interactions in an international case-control study. Int J Cancer.

[37] Montero JC, Ocaña A, Abad M, Ortiz-Ruiz MJ, Pandiella A, Esparís-Ogando A (2009). Expression of Erk5 in early stage breast cancer and association with disease free survival identifies this kinase as a potential therapeutic target. PLoS One.

[38] Colleoni M, Rotmensz N, Robertson C, Orlando L, Viale G, Renne G (2002). Very young women (<35 years) with operable breast cancer: features of disease at presentation. Ann Oncol.

